# Musculoskeletal pain in people with and without type 2 diabetes in Taiwan: a population-based, retrospective cohort study

**DOI:** 10.1186/s12891-015-0819-4

**Published:** 2015-11-20

**Authors:** Lee-Wen Pai, Chin-Tun Hung, Shu-Fen Li, Li-Li Chen, Yueh- Chin Chung, Hsin-Li Liu

**Affiliations:** Department of Public Health, China Medical University, Taichung, Taiwan; Department of Nursing, Central Taiwan University of Science and Technology, No.666, Buzih Road, Beitun District Taichung City, 40601 Taiwan; Department of Healthcare Administration, Central Taiwan University of Science and Technology, Taichung, Taiwan; School of Nursing, China Medical University, No.91 Hsueh-Shih Road, Taichung, 40402 Taiwan; Department of Nursing, China Medical University Hospital, No.91 Hsueh-Shih Road, Taichung, 40402 Taiwan

**Keywords:** Type 2 diabetes, Musculoskeletal pain, Incidence

## Abstract

**Background:**

Musculoskeletal pain in people with type 2 diabetes is a common issue even to this day. The study aimed to explore the 10-year cumulative incidence of musculoskeletal pain, the mean number of doctor visits for musculoskeletal pain, and the mean number of doctor visits for musculoskeletal pain by location in people with type 2 diabetes, compared with respective values for people without diabetes.

**Methods:**

The study utilized a population-based retrospective cohort study design. The subjects were randomly obtained from the Taiwan National Health Insurance Research Database. The diabetic group included 6586 people with type 2 diabetes aged 18–50 years, while the non-diabetic group consisted of 32,930 age- and sex-matched people. Based on the medical records of individuals with musculoskeletal pain in the two groups from 2001 to 2010, the 10-year cumulative incidence of musculoskeletal pain, the mean number of doctor visits for musculoskeletal pain, and the mean number of doctor visits for musculoskeletal pain by location were calculated and compared, with the aim of identifying differences between the two groups.

**Results:**

Showed that people in the diabetic group had a higher 10-year cumulative incidence of and a higher mean number of doctor visits for musculoskeletal pain than the non-diabetic group (*p* < 0.05). The relative risk (RR) of the 10-year cumulative incidence of musculoskeletal pain in the two groups was the highest (RR = 1.39) for people between 30 and 39 years of age. The mean number of doctor visits for musculoskeletal pain by location was significantly different between the two groups. However, the mean number of doctor visits for limb pain registered the largest difference between the two groups.

**Conclusion:**

People with type 2 diabetes aged 18–50 years had a higher 10-year cumulative incidence of and a higher mean number of doctor visits for musculoskeletal pain than the non-diabetic group. Musculoskeletal pain might directly or indirectly interfere with or decrease the physical activity levels of people with diabetes. Therefore, it is important to detect and treat musculoskeletal pain early in order to promote physical activity and optimize blood sugar control.

## Background

Diabetes is one of the leading causes of mortality globally [1]. According to World Health Organization statistics published in 2013, approximately 347 million people worldwide suffer from diabetes, and by 2030 diabetes will be the seventh leading cause of death [2]. In Taiwan, diabetes has remained a serious public health concern ever since 1984, when diabetes rose to the tenth leading causes of death [3].

Type 2 diabetes mellitus is a disease that can lead to a progressive insulin secretory defect on top of insulin resistance [4]. Many studies have indicated that its incidence is related to age [5], obesity [6], family inheritance [7], impaired glucose metabolism [8], and a sedentary lifestyle [9]. People who do not manage their blood glucose levels well often suffer from persistent hyperglycemia, leading to an increase in the production of advanced glycation end products, and causing stiffness of connective tissue and subsequent aggravated musculoskeletal pain [10]. Previous studies have indicated that the causes of increased musculoskeletal pain in people with diabetes are probably related to vascular insufficiency [11], neuropathy [12], decreased insulin-like growth factor 1 [13], accelerated osteoporosis [13], obesity [14], sedentary lifestyle [15] and other factors. Moreover, musculoskeletal pain can directly or indirectly impede or decrease the physical activity levels of people with diabetes. When people with diabetes cannot maintain ideal levels of physical activity, it can affect blood sugar control, causing complications and a decline in quality of life. Therefore, maintaining a proper level of physical activity is a critical part of treatment for people with diabetes, rendering it of equal importance to diet and medicine. Therefore, it is necessary to diagnose and investigate musculoskeletal pain in people with diabetes as early as possible in order to provide proper treatment.

Existing literature on the topic [16–19] indicates a paucity of long-term follow-up research centered on people with type 2 diabetes suffering from musculoskeletal pain. In fact, most of the existing studies used self-report data from patients. Taiwan has enjoyed a complete national health insurance system since its establishment in 1986, and the proportion of the population who are insured is higher than 99 % [20]. The database utilized for this study, the massive and precise Taiwanese National Health Insurance Research Database, was compiled based on diagnostic information from insurance payment records from the National Health Insurance of Taiwan. The objective of this study was to explore the 10-year cumulative incidence of musculoskeletal pain, the mean number of doctor visits for musculoskeletal pain, and the mean number of doctor visits for musculoskeletal pain by location in people with type 2 diabetes aged 18–50 years in Taiwan from 2001 to 2010, and to compare these results with respective values for people without diabetes. This study provides empirical evidence and practical considerations that will allow healthcare professionals to improve the clinical treatment and care for people with type 2 diabetes suffering from musculoskeletal pain. It is important to detect and treat musculoskeletal pain early in order to promote physical activity and optimize blood sugar control.

## Methods

From the Taiwanese National Health Insurance Research Database, one million people were randomly drawn as samples and a 10-year retrospective cohort study was conducted. The subjects included individuals with type 2 diabetes (diabetic group) who were newly diagnosed in 2001 and individuals without type 2 diabetes (non-diabetic group).

The Taiwanese National Health Insurance Research Database recorded the individuals’ demographic data and outpatient medical diagnostic codes. First, all people with type 2 diabetes with a Taiwan National Health Insurance expense application diagnostic code 250.0 or A code: A181 (i.e. type 2 diabetes without complications) and who had more than three outpatient records in 2001 were included. Excluded were people having a medical record of diabetes and musculoskeletal pain within 365 days before the first diabetic medical record in 2001. The locations of musculoskeletal pain were broadly categorized as shoulder and neck pain, limb (including arms, hands, and knees) pain, lumbar pain, back pain or pelvis pain, which were referencing categories used by other studies [16]. Musculoskeletal pain in patients with the following Taiwan National Health Insurance payment ICD-9-CM diagnostic codes were included: 719.41 (pain in shoulder joint), 723.1 (cervicalgia), 719.42 (pain in upper arm joint), 719.43 (pain in forearm joint), 719.44 (pain in hand joint), 719.46 (pain in lower leg joint), 719.47 (pain in ankle and foot joint), 729.1 (myalgia and myositis), 729.5 (pain in limb), 719.45 (pain in pelvic region and thigh joint), 724.2 (low back pain), 724.3 (sciatica) and 724.5 (backache). Qualified clinicians classified the patients according to diagnostic codes.

The age range in this study was 18–50 years, with the upper limit based on the threshold for elderly status being over 50 years in some countries [21]. According to Wilkie et al., who conducted a three- and six-year population-based prospective study based on 2949 individuals over 50 years of age, the incidence for individuals over 50 years having musculoskeletal pain is related to the healthy aging index. Individuals with a lower index are more likely to have musculoskeletal pain, and the index decreases with increase in age. Therefore, there is a correlation between the health of elderly people over 50 years and the incidence of musculoskeletal pain [22]. With this in mind, and also taking into consideration that widespread pain is a common problem for elderly people and may be influenced by many factors [23], we excluded research subjects over the age of 50 to avoid the confounding effects of age and health on the analysis. In addition, the growth and development of children is different from adults, so we excluded children under 18 years old. Therefore based on these criteria, 6586 eligible subjects were selected for the diabetic group.

The inclusion criteria for subjects in the non-diabetic group were: no medical records related to diabetes from 2000 to 2010, no outpatient records related to musculoskeletal pain in 2000, and a record of survival from 2001 to 2010. Thus 881,968 potentially eligible individuals were identified. After the survival of individuals in both groups in 2010 was confirmed, the individuals were matched according to their age and sex at a ratio of 1:5 (diabetic group: non-diabetic group), and 32,930 individuals were selected for the non-diabetic group.

Next, the use of all categories of outpatient service in both groups was evaluated. Finally, the use of outpatient service for musculoskeletal pain in both groups each year from 2001 to 2010 was examined. The medical records due to musculoskeletal pain from 2001 to 2010 in the two groups according to diagnostic codes were tracked, from which the 10-year cumulative incidence of musculoskeletal pain, the mean number of doctor visits for musculoskeletal pain, and the mean number of doctor visits for musculoskeletal pain by location were calculated. Data from the two groups were then compared to uncover differences.

The 10-year cumulative incidence of musculoskeletal pain was calculated as the number of new patients with musculoskeletal pain from 2001 to 2010 divided by the total number of individuals in the group. The mean number of doctor visits for musculoskeletal pain in each year was calculated as the sum of the doctor visits of every individual due to musculoskeletal pain in each year divided by the total number of individuals in the group. The mean number of doctor visits for musculoskeletal pain from 2001 to 2010 was calculated as the sum of the doctor visits of every individual due to musculoskeletal pain from 2001 to 2010 divided by the total number of individuals in the group multiplied by 10. The mean number of doctor visits for musculoskeletal pain by location was calculated as the sum of the doctor visits of every individual for each pain location from 2001 to 2010 divided by the total number of individuals in the group multiplied by 10. Chi-square tests were used to assess differences in the 10-year cumulative incidence of musculoskeletal pain between the two groups. Relative risk (RR) represents the ratio of the 10-year cumulative incidence of musculoskeletal pain in the diabetic group versus the non-diabetic group. Additionally, data for the mean number of doctor visits for musculoskeletal pain and the mean number of doctor visits for musculoskeletal pain by location between the two groups were expressed as mean ± SD (standard deviation). The significance in differences in the medians of two groups was assessed by the Mann–Whitney *U* test.

### Ethical considerations

All subject data were encrypted using the same encryption algorithm to cross-link the data while protecting the privacy of the patients. This study protocol was approved by the institutional review board (IRB) of China Medical University Hospital (protocol #CMUH103-REC1-088).

## Results

This study included 39,516 individuals as subjects: 6586 individuals in the diabetic group and 32,930 individuals in the non-diabetic group. The diabetic group consisted of 338 (5.1 %) individuals from 18–29 years of age, 1338 (20.3 %) from 30–39 years of age, and 4910 (74.6 %) from 40–50 years of age, with 3917 (59.5 %) males and 2669 (40.5 %) females. The non-diabetic group consisted of 1690 (5.1 %) individuals from 18–29 years of age, 6690 (20.3 %) from 30–39 years of age, and 24,550 (74.6 %) people from 40–50 years of age, with 19,585 (59.5 %) males and 13,345 (40.5 %) females. In both diabetic and non-diabetic groups, the 40–50 age group had the highest number of subjects, followed by the 30–39 age group, and the 18–29 age group; the percentage of males was higher than that of females in both groups.

### Comparison of the 10-year cumulative incidence of musculoskeletal pain

The diabetic group had a higher 10-year cumulative incidence of musculoskeletal pain than the non-diabetic group for all age groups and in both females and males (*p* < 0.05), as illustrated in Table 1. The RR for all age groups and genders was > 1. Furthermore, the RR was the highest in the 30–39 age group, lowest in the 18–29 age group, and higher in females than in males.

**Table 1:** 10-year cumulative incidence of musculoskeletal pain and analysis in both groups (*N* = 39,516)

	10-year cumulative incidence (%)^a^		*χ* ^2^	RR^C^	95 % CI of RR
	Diabetic group	Non-diabetic group			
Age					
18–29	13.61	12.01	0.6675	1.13	1.05-1.22
30–39	20.18	14.51	27.3821^b^	1.39	1.31-1.48
40–50	23.22	19.87	28.2924^b^	1.17	1.11-1.23
Sex					
Male	19.07	16.31	17.7761^b^	1.17	1.10-1.24
Female	26.56	21.40	34.2590^b^	1.24	1.18-1.30

^a^10-year cumulative incidence was calculated as the number of new patients with musculoskeletal pain from 2001 to 2010 divided by the total number of individuals in that group

^b^ *p* < 0.001

^c^ *RR* relative risk

### Comparison of the mean number of doctor visits for musculoskeletal pain

Table 2 shows the mean number of doctor visits for musculoskeletal pain for individuals aged 18–50 years in the diabetic and non-diabetic groups from 2001 to 2010. Except for 2006, 2007 and 2010, the diabetic group had significantly higher annual and 10-year mean number of doctor visits for musculoskeletal pain than the non-diabetic group (*p* < 0.05).

**Table 2:** Comparison of the mean number of doctor visits for musculoskeletal pain in both groups (*N* = 39,516)

Year	Diabetic group	Non-diabetic group	*Z*	*p* ^*c*^
	n: 6586	n: 32930		
	Mean^a^ ± SD	Mean^a^ ± SD		
2001	0.51 ± 1.55	0.35 ± 1.23	−3.461	0.001
2002	0.69 ± 2.27	0.48 ± 1.63	−2.495	0.013
2003	0.74 ± 2.48	0.50 ± 1.67	−2.681	0.007
2004	0.72 ± 2.04	0.53 ± 1.75	−2.607	0.009
2005	0.69 ± 1.92	0.55 ± 1.85	−2.057	0.040
2006	0.58 ± 1.79	0.53 ± 1.82	−0.818	0.413
2007	0.60 ± 1.98	0.49 ± 1.74	−1.808	0.071
2008	0.75 ± 2.40	0.46 ± 1.75	−3.504	0.001
2009	0.77 ± 2.37	0.50 ± 1.94	−1.987	0.047
2010	0.77 ± 2.54	0.48 ± 1.60	−1.901	0.057
2001-2010	0.67 ± 2.12^b^	0.48 ± 1.68^b^	−7.402	0.000

^a^The mean number of doctor visits for musculoskeletal pain in each year was calculated as the sum of the doctor visits of every individual due to musculoskeletal pain in each year divided by the total number of individuals in the group

^b^The mean number of doctor visits for musculoskeletal pain from 2001 to 2010 was calculated as the sum of the doctor visits of every individual due to musculoskeletal pain from 2001 to 2010 divided by the total number of individuals in the group multiplied by 10

^c^ *p* value was calculated by Mann–Whitney *U* test

*SD* standard deviation

### Comparison of the mean number of doctor visits for musculoskeletal pain by location

From 2001 to 2010, the mean number of doctor visits for musculoskeletal pain in the neck and shoulders, limbs, lumbar spine, back and pelvis was higher in the diabetic group than the non-diabetic group (*p* < 0.001) (see Table 3). In relation to the location of musculoskeletal pain, lumbar spine, back and pelvis pain accounted for the highest mean number of doctor visits in both groups. The difference in this value was the highest for limb pain between the two groups.

**Table 3:** Comparison of the mean number of doctor visits for musculoskeletal pain by location in both groups (*N* = 39,516)

	Diabetic group	Non-diabetic group	*Z*	*p* ^b^
	n: 6586	n: 32,930		
	Mean^a^ ± SD	Mean^a^ ± SD		
Neck and shoulders	0.07 ± 0.64	0.04 ± 0.39	2.803	0.005
Limbs	0.27 ± 0.25	0.17 ± 0.91	7.405	0.000
Lumbar, back and pelvis	0.35 ± 1.42	0.28 ± 1.28	4.157	0.000

^a^ The mean number of doctor visits for musculoskeletal pain by location was calculated as the sum of the doctor visits of every individual for each pain location from 2001 to 2010 divided by the total number of individuals in that group multiplied by 10

^b^ *p* value was calculated by Mann–Whitney *U* test

*SD* standard deviation

### Utility

The study found that the 10-year cumulative incidence of musculoskeletal pain and the mean number of doctor visits for musculoskeletal pain were higher in individuals with type 2 diabetes aged 18–50 years than in non-diabetic individuals. Assessing for musculoskeletal pain in people with type 2 diabetes is necessary, and early detection and intervention might help to maintain ideal physical activity for this population.

## Discussion

The study found that the 10-year cumulative incidence of musculoskeletal pain and the mean number of doctor visits for musculoskeletal pain were higher in individuals with type 2 diabetes aged 18–50 years than in non-diabetic individuals. In addition, the RR for musculoskeletal pain for the diabetic group compared with the non-diabetic group was greater than 1, which was the highest for individuals in the 30–39 age group. The mean number of doctor visits for musculoskeletal pain in the limbs showed the highest difference between the two groups. The results provide medical professionals information about the incidence of musculoskeletal pain in people with type 2 diabetes. Musculoskeletal pain directly or indirectly interferes with or decreases the physical activity levels of people with diabetes [24]. A systematic assessment of the musculoskeletal system of people with type 2 diabetes should be performed early in the diabetic disease course early and proper detection of musculoskeletal pain [25].

This study found that the mean number of doctor visits for musculoskeletal pain for people with type 2 diabetes in Taiwan within the 10-year period did not increase with the progression of diabetes. This is different from what was observed in a study by Zamani et al., which suggested that the risk of musculoskeletal pain would increase with time over 15 years [17]. This disparity may be because our study assessed the phenomena over a different time span. Additionally, in the years 2006, 2007 and 2010, the mean number of doctor visits for musculoskeletal pain was not significantly different between the diabetic and the non-diabetic groups. This finding might be related to improvement in diabetes treatment and care in Taiwan during this three-year period. Previous studies [26] suggested that persistent hyperglycemia may increase the incidence of musculoskeletal pain. During this three-year period, Taiwan had promoted multiple policies on diabetes prevention and treatment [27]. Further assessment is necessary to determine conclusively whether these policies had successfully motivated people with diabetes to follow an active lifestyle and improve their self-management of blood sugar control, which in turn led to a decrease the mean number of doctor visits for musculoskeletal pain.

This study demonstrated that the RR for musculoskeletal pain in people with diabetes was higher for females than males and the 10-year cumulative incidence of musculoskeletal pain for both the diabetic and non-diabetic groups was higher in females than males. This is consistent with findings from other studies [16]. Ramírez-Maestre and Esteve investigated 400 people with chronic spinal pain and their results showed that females had higher pain intensity and pain anxiety than males [28]. Racine et al. reviewed 172 studies from 1998 to 2008 using a systematic literature review, and their results supported the notion that pressure pain thresholds were lower in females than males [29]. The current study analyzed the 10-year cumulative incidence and the mean number of doctor visits for musculoskeletal pain based on medical records. Further study in the future is needed to elucidate whether the mean number of doctor visits for musculoskeletal pain in females is due to higher pain intensity, higher pain anxiety, and lower pressure pain thresholds among females compared to in males, or if the 10-year cumulative incidence for musculoskeletal pain is indeed higher for females with type 2 diabetes than for males with the same condition. Other possible factors that might influence individuals’ motivation for their first doctor visit as well as the mean number of doctor visits for musculoskeletal pain include: age, availability of doctors, and accessibility of medical resources, among others [30]. For this study samples were randomly drawn and individuals were matched according to age and sex to minimize the influence of the other possible factors on the research outcomes.

Among all locations of musculoskeletal pain, the two groups exhibited the highest difference for limb pain. This may be related to long-term poor blood sugar control, which can cause damage in the bones and nerves of the limbs [31, 32], and subsequently increase the frequency of musculoskeletal pain in this location. The mean number of doctor visits for lumbar spine, back, or pelvis pains was the highest, which is consistent with the findings of Eivazi [33]. Molsted et al. showed that pain in the lower back is related to high body mass index, sedentary lifestyle and reduced physical function. In addition, pain in the hands, knees, or hip is related to high body mass index [16]. Therefore, high body mass index, sedentary lifestyle, reduced physical function, diseases of the bones and nerves of the limbs, and other factors may contribute to an increase in musculoskeletal pain in the lumbar spine, back, limbs, and pelvis for people with diabetes. Further studies may help address the relative contributions of these related factors.

The power of a study is the largest when the pairing ratio of 1:1 given the sample size is fixed. One way to increase the power of a study is to increase the number of controls when the investigator cannot increase number of cases. In this study, since there were not as many diabetic subjects as non-diabetic subjects, the 1:5 pairing ratio was used to increase the statistical power. This kind of pairing method will not create added bias on the results because each study subject of case and control groups was randomly selected from his/her population. The limitation of this method is that the precision of estimates could only be increased for control group, not for the case group [34]. The age- and sex-matched sampling method eliminated confounding effects of age and sex on the incidence of musculoskeletal pain in the results [11, 22]. The information was collected from actual medical records to avoid imprecision due to imperfect memory, which is a risk when using self-report data from patients. Since people with diabetes under 18 and over 50 years of age were excluded, the results of this study are limited to people with type 2 diabetes within this age range. Previous studies showed that the causes of an increase in people with diabetes having musculoskeletal pain may relate to hyperglycemia [32], obesity [14], physical functions and other factors [12]. However, because of limitations surrounding access to the National Health Insurance Database, related variables, such as blood sugar level, body mass index, weight, or physical functions could not be controlled or adjusted. Therefore, that future research studies should control and adjust for other factors that may cause musculoskeletal pain in order to avoid complications in the interpretation of relative musculoskeletal pain incidence in diabetic versus non-diabetic people in Taiwan.

## Conclusion

The study demonstrates that people with type 2 diabetes aged 18–50 years had a higher 10-year cumulative incidence of musculoskeletal pain and a higher mean number of doctor visits for musculoskeletal pain than those without diabetes. The mean number of doctor visits for musculoskeletal pains by locations was significantly different in both groups. In relation to pain locations, musculoskeletal pain in the lumbar spine, back, and pelvis accounted for the highest mean number of doctor visits for musculoskeletal pain in both groups. However, the mean number of doctor visits for musculoskeletal pain in the limbs registered the largest difference between the two groups. Therefore, professionals should pay special attention to musculoskeletal pain issues when treating people with type 2 diabetes. It is important to detect and treat musculoskeletal pain early in order to promote physical activity and optimize blood sugar control.

## Abbreviations

RR: Relative risk
SD: Standard deviation
